# Distinct Clinical and Prognostic Features of Myelodysplastic Syndrome in Patients from the Middle East, North Africa, and Beyond: A Systemic Review

**DOI:** 10.3390/jcm12082832

**Published:** 2023-04-12

**Authors:** Amal Al-Haidose, Mohamed A. Yassin, Muna N. Ahmed, Hasna H. Kunhipurayil, Asrar A. Al-Harbi, Musheer A. Aljaberi, Saddam A. Abbasi, Shahram Kordasti, Atiyeh M. Abdallah

**Affiliations:** 1 Department of Biomedical Sciences, College of Health Sciences, QU Health, Qatar University, Doha 2713, Qatar; 2 Medical Oncology Department-Hematology Section, National Centre for Cancer Care and Research, Hamad Medical Corporation, Doha 3050, Qatar; 3 Al-Rayan Colleges, Al Madinah Al Munawwarah 42541, Saudi Arabia; 4 Faculty of Medicine & Health Sciences, Taiz University, Taiz 6803, Yemen; 5 Statistics Program, Department of Mathematics, Statistics, and Physics, College of Arts and Sciences, Qatar University, Doha 2713, Qatar; 6 Statistical Consulting Unit, College of Arts and Science, Qatar University, Doha 2713, Qatar; 7 School of Cancer and Pharmaceutical Science, King’s College London, London WC2R 2LS, UK; 8 Haematology Department, Guy’s and St. Thomas NHS Trust, London SE1 9RT, UK

**Keywords:** myelodysplastic syndrome, epidemiology, cytogenetics, prognosis, Asia, North Africa, MENA

## Abstract

Myelodysplastic syndrome (MDS) describes a group of bone marrow malignancies with variable morphologies and heterogeneous clinical features. The aim of this study was to systematically appraise the published clinical, laboratory, and pathologic characteristics and identify distinct clinical features of MDS in the Middle East and North Africa (MENA) region. We conducted a comprehensive search of the PubMed, Web of Science, EMBASE, and Cochrane Library databases from 2000 to 2021 to identify population-based studies of MDS epidemiology in MENA countries. Of 1935 studies, 13 independent studies published between 2000 and 2021 representing 1306 patients with MDS in the MENA region were included. There was a median of 85 (range 20 to 243) patients per study. Seven studies were performed in Asian MENA countries (732 patients, 56%) and six in North African MENA countries (574 patients, 44%). The pooled mean age was 58.4 years (SD 13.14; 12 studies), and the male-to-female ratio was 1.4. The distribution of WHO MDS subtypes was significantly different between MENA, Western, and Far East populations (*n* = 978 patients, *p* < 0.001). More patients from MENA countries were at high/very high IPSS risk than in Western and Far East populations (730 patients, *p* < 0.001). There were 562 patients (62.2%) with normal karyotypes and 341 (37.8%) with abnormal karyotypes. Our findings establish that MDS is prevalent within the MENA region and is more severe than in Western populations. MDS appears to be more severe with an unfavorable prognosis in the Asian MENA population than the North African MENA population.

## 1. Introduction

Myelodysplastic syndrome (MDS) describes a group of bone marrow malignancies characterized by cytopenia and abnormal clonal hematopoiesis. MDS commonly affects older individuals, with a median age of onset of 71 years (range 65 to 80 years) reported in patients from Western countries [1]. MDS is heterogeneous, and thus treatment protocols vary between patients [2,3]. The disease is more common in males than females and also common in individuals with prior exposure to cytotoxic, radiation, and radioiodine therapies [4]. There are at least 4–7 new cases of MDS per 100,000 persons in the United States and Western Europe each year, and the incidence is increasing dramatically [5]. Most MDS patients present with normal or hypercellular bone marrow, but ~15% have hypoplastic marrow. Hypoplastic MDS is more common in Middle Eastern countries, and these patients respond poorly to treatment. Up to 30% of MDS patients are at risk of developing acute myeloid leukemia (AML) [6], with age under 40 years being the main risk factor for leukemic transformation [7]. Mirroring the phenotypic heterogeneity, MDS patients have a highly variable prognosis, with a reported median survival of anywhere between less than six months to over five years [8]. Although the Surveillance, Epidemiology, and End Results (SEER) program collected statistical data on MDS and other cancers, MDS is still known to be underreported due to a lack of uniform classification criteria, changing coding guidelines, and limited reporting by outpatient clinics [9].

The initial diagnosis of MDS is based on the observation of constant cytopenia (hemoglobin < 100 g/L; absolute neutrophil count < 1.8 × 10^9^/L; platelet count < 100 × 10^9^/L), dysplasia in any hematopoietic lineage, blast cells, and cytogenetic abnormalities [4]. MDS risk (or prognosis) can be assessed using different prognostic scoring systems including the French-American-British (FAB) classification, published in 1976; the International Prognostic Scoring System (IPSS), published in 1997 and revised (IPSS-R) in 2012; and the World Health Organization (WHO) classification-based Prognostic Scoring System (WPSS) [10]. The WPSS classification and IPSS-R scoring system are routinely updated and internationally accepted.

The MENA (Middle East and North Africa) region represents many countries but, for the purposes of this review, we follow Michael and Staley’s 2007 definition of the Arab countries (Algeria, Bahrain, Djibouti, Egypt, Iraq, Jordan, Kuwait, Lebanon, Libya, Mauritania, Morocco, Oman, Palestine, Qatar, Saudi Arabia, Somalia, Sudan, Syria, Tunisia, United Arab Emirates, and Yemen) in addition to Turkey and Iran [11]. The epidemiological data on MDS in MENA countries are scarce. A few studies have reported the age of onset is younger and the disease more likely to harbor high-risk cytogenetic aberrations in MDS patients in the region. For example, in a recent Saudi Arabian study, the mean age of diagnosis was 50 (±18) years [12]. Interestingly, the data showed that Saudi patients have more aggressive disease at presentation than Western patients, with over 50% of patients classified as having “poor” and “very poor” prognoses following the IPSS-R classification. However, a study from Morocco reported a mean age of diagnosis of 65.7 (±14) years, with the prognosis of 83% of patients classified as “very good” and “good” [13]. Therefore, the demographic and clinical features of MDS in patients in the MENA region remain uncertain.

Most of the literature on the characteristics of MDS (such as clinical presentation, disease severity, cytogenetic abnormalities and mutations, and disease classification) is from Western populations, which may not be generalizable to other populations in clinical practice. Therefore, here we aimed to systematically appraise peer-reviewed publications on the characteristics of MDS in the MENA region and compared them with the global data. To our knowledge, this is the first systematic review reporting MDS features in the MENA region, and we expect it to help clinical practice in terms of prognostication and treatment protocols in the MENA region and, by understanding the distinct features in different populations, beyond.

## 2. Materials and Methods

### 2.1. Search Strategy

Three reviewers performed a systematic literature search of the PubMed, Web of Science, EMBASE, and Cochrane Library databases to identify published data on the clinical features of MDS in MENA populations. The search terms were a combination of the name of each country together with the keywords of “MDS” or “myelodysplastic syndrome”. The same search methods were applied to all four databases. We focused on WPSS and IPSS-R prognostic outcomes and excluded articles classified according to FAB. Therefore, we limited our search to publications published between 1 January 2000 and 30 November 2022. Our study adhered to and followed the PRISMA-2020 statement guidelines in the performing and reporting of the systemic review; however, we did not undertake the systematic review registration [14].

### 2.2. Geographical Definitions

We divided the MENA countries into two groups: the Asian MENA countries and the North African MENA countries. Studies included in the Asian MENA region were from Iran, Lebanon, Oman, Saudi Arabia, and Turkey, and studies in the African MENA region were from Algeria, Egypt, and Morocco. To obtain a deep understanding of MDS in the MENA region, we compared data derived from MENA countries with Western and Far East data reported by Jiang et al. 2021 [15]. Therefore, studies from the USA, Netherlands, and Sweden and from the Far East, i.e., Japan, Korea, China, and Taiwan were included for international comparative analysis.

### 2.3. Study Selection

**Figure 1:**
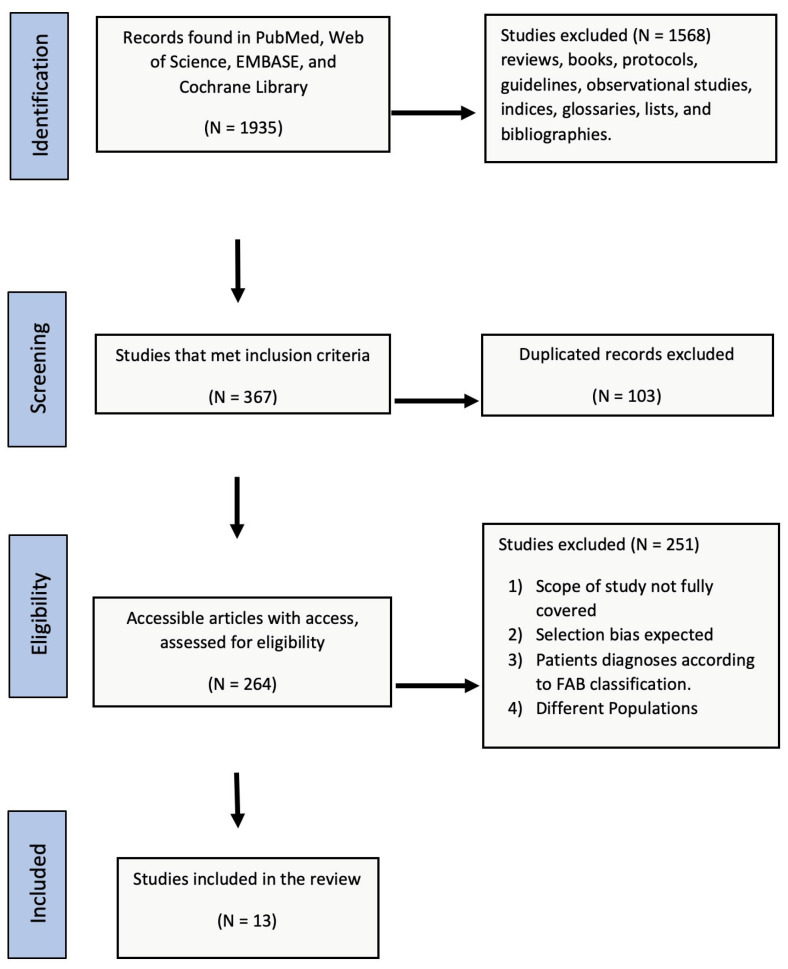
PRISMA flowchart of the literature review.

Two researchers separately reviewed the retrieved records. In cases of disagreement, consensus was reached with a third author. The total number of records identified in each database was recorded. Records were restricted to research articles and reviews, books, protocols, guidelines, and observational studies, and other elements (e.g., indices, glossaries, lists, and bibliographies) were excluded. After removing duplicates, the remaining records were checked for eligibility for primary selection through their titles and abstracts for diagnoses based on IPSS-R or WPSS classifications and study populations in the MENA region. Records were excluded if they were diagnosed using the FAB classification or if they were conducted in another study population. To collect relevant, consistent evidence, there was a round of a secondary selection to select records by screening the entire full text (Table 1). Two investigators evaluated the studies, and inconsistencies were resolved by a third (senior) reviewer by agreement. The screened and selected records are reported in a PRISMA flow chart (Figure 1).

### 2.4. Quality Assessment

Two reviewers independently evaluated the quality of the 13 included studies and overall risk of bias using the QUADAS-2 (Quality Assessment of Diagnostic Accuracy Studies-2) tool. This tool, which evaluates the risk of bias and applicability of diagnostic studies, is recommended by The Cochrane Collaboration and was implemented in RevMan v5 (Oxford, UK) [16,17]. QUADAS-2 rates patient selection, the index test, the reference standard, and flow and time. The possibility of bias is assessed using signaling questions. Study applicability was determined by whether the study addressed the review question. Each domain was rated as “low risk of bias”, “high risk of bias”, or “unclear risk of bias”. The first three domains were also rated with respect to applicability. If the answer to any of the signaling questions was “no”, the study was rated as “high risk”. The study was “low risk” if all signaling questions were answered with a “yes” and “unclear risk” if the answer to one or more of the signaling questions was “unclear”. A third opinion was sought in the event of disagreement.

### 2.5. Data Extraction and Statistical Analysis

Data were extracted from the tables and/or text of eligible articles and recorded in a standard Microsoft Excel spreadsheet. Two researchers reviewed the collected data independently, and any discrepancies were discussed with a senior investigator. Extracted variables included gender, age, mean, and standard deviation (SD) of clinical laboratory findings (hemoglobin (Hb), platelet count, white blood cell (WBC) count, cytopenia, and bone marrow blasts), disease classification based on WPSS, and disease prognostic risk scores based on the IPSS-R. In addition, data on cytogenetic abnormalities were collected where available. Quantitative variables such as age, Hb, WBC, and platelet counts were recorded as mean ± SD. If the mean age was not provided and other statistical values were given, we used the method described by McGrath et al. to estimate the sample mean from the available values (https://smcgrath.shinyapps.io/estmeansd/, accessed on 1 September 2022) [18]. For the pooled mean calculation, we used an online tool (https://home.ubalt.edu/ntsbarsh/business-stat/otherapplets/Pooled.htm, accessed on 1 September 2022). Graphical representations and statistical analyses were performed in R. The analysis included testing for the equality of means and equality of proportions. Categorical data such as gender, disease classification, prognostic risk, and cytogenetic abnormalities were presented as frequencies and percentages. We used ANOVA and post hoc Tukey HSD tests to test the equality of means. Moreover, to check the equality of proportions, we used the chi-squared test and a pos hoc test for pairwise proportion comparisons. A *p*-value ≤ 0.05 was considered statistically significant.

## 3. Results

### 3.1. Identification and Selection of Records

A total of 1935 articles were retrieved from all databases, and 1568 studies were excluded due to being reviews, books, protocols, guidelines, observational studies, indices, glossaries, lists, or bibliographies. A total of 103 duplicate entries were excluded to leave 264 records for primary and secondary screening by title, abstract, and then full text according to the PICO scheme for eligibility assessed against the inclusion and exclusion criteria. There were 251 records which were excluded due to expected selection bias, diagnosis according to the FAB classification, and/or being conducted in non-MENA populations. The remaining 13 articles were included for complete analysis (Supplementary Table S1).

### 3.2. Quality of the Eligible Studies

**Figure 2:**
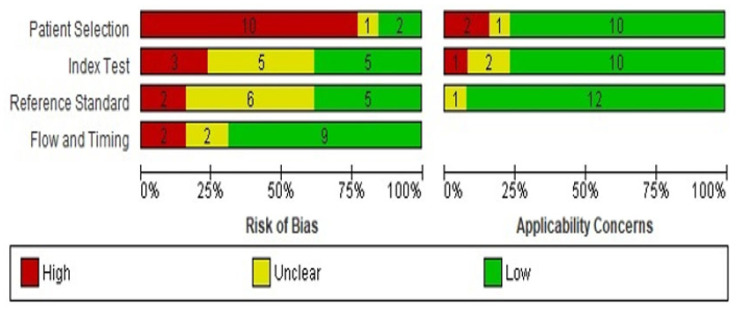
Summary of the risk of bias and applicability concerns.

Figure 2 shows the evaluation of the risk of bias and the recurrent sources of bias in the four domains of QUADAS-2. The highest risk of bias (80%) was in the first domain, patient selection, where participants were preselected and 11 studies were retrospective and 2 were prospective. In the second domain (index test), 20% of studies were rated as having a high risk of bias, whereas 40% had an unclear risk and 40% a low risk of bias, where a clear description of the diagnostic threshold and a standardized procedure for the index test were provided. There was a high risk of bias in the reference standard in 15% of studies, which means that the interpretation of the reference standard led to bias or there was unclear evidence regarding the reference standard. For flow and timing, 70% of evaluated studies were rated as having a low risk of bias because, in most studies, there was an appropriate interval between the index test(s) and the reference standard. Regarding applicability, the patient selection, index test, and reference standard domains were applicable to the review question and for most there was low concern about applicability for the three domains.

### 3.3. Narrative Systematic Review

#### 3.3.1. Study Characteristics

Thirteen independent studies were published between 2000 and 2021, representing 1306 patients diagnosed with MDS. The median number of patients per study was 85 (range 20 to 243). Seven studies were performed in the Asian MENA region (732 patients, 56%) and six were conducted in the North African MENA region (574 patients, 44%) (Table 2). Secondary MDS was reported by only five studies: one study from North African MENA and four studies from Asian MENA. Overall, 40 out of 426 patients (9.4%) had secondary MDS.

#### 3.3.2. Gender and Age

**Figure 3:**
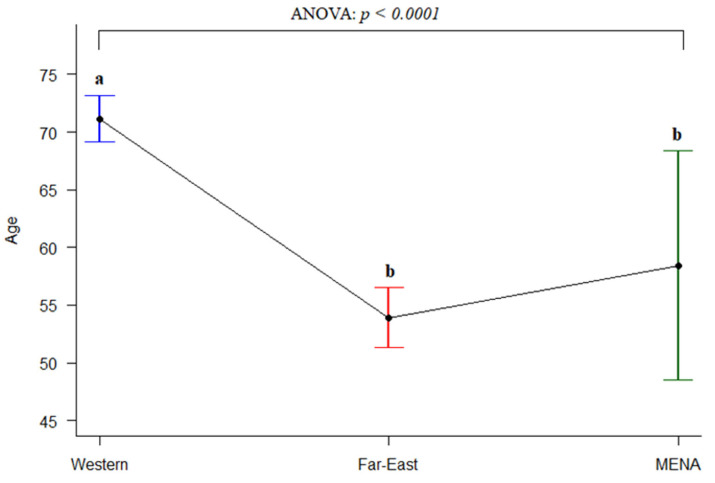
Comparison of the average age of onset for Western, Far East, and MENA populations (Western and Far East data were taken from [28,29,30,31,32,33,34,35]). Letters show significant differences: the same letter b is not significant, whereas a denotes statistically significant differences between groups.

MDS patients were predominantly male (male to female ratio 1.4, range 0.67 to 2.68), especially in the Asian MENA population (1.5 vs. 1.3 in the North African MENA; Table 2). The mean age was available in 12 studies, and the overall mean was 58.4 years with a pooled standard error of 5.1 (SD 13.14). No significant difference was observed in the average age between Asian MENA and North African MENA populations. We further compared the average age of onset of MENA, Western [28,29,30,31], and Far East populations [32,33,34,35], which were 58.42, 71.15, and 53.92, respectively. Figure 3 displays the interval plot and ANOVA test results for the average age of onset of MDS in the three groups (Western, Far East, and MENA; significantly different, *p* < 0.0001). In post hoc tests, the averages of the Far East and MENA populations were not significantly different but both were significantly different to that of the Western population (Figure 3).

#### 3.3.3. Hematological and Laboratory Data

The mean hemoglobin (Hb) was available in ten studies representing 964 patients. The pooled mean Hg was 8.4 g/dL. The lowest mean Hb was 6.8 g/dL and the highest 10.3 g/dL. The mean platelet count was reported in nine studies representing 870 patients and ranged from 77 to 325 × 10^3^/μL; the mean platelet count was 90 × 10^3^/μL. The mean WBC count was reported in six studies representing 694 patients, and the mean pooled WBC was 5 × 10^3^/μL. 

Data on cytopenia at presentation were only available for 359 patients in five studies. Unicytopenia was seen in 137 patients (38.2%), bicytopenia in 99 patients (27.6%), pancytopenia in 122 patients (34%), and no cytopenia in only 1 patient (0.3%). Bone marrow blasts were reported in 191 patients in four studies. Most patients (70.2%) had <5% bone marrow blasts, followed by 10% blasts in 35 (18.3%) and 15% blast in 22 patients (11.5%) (Table 3).

#### 3.3.4. Patient Classification

**Figure 4:**
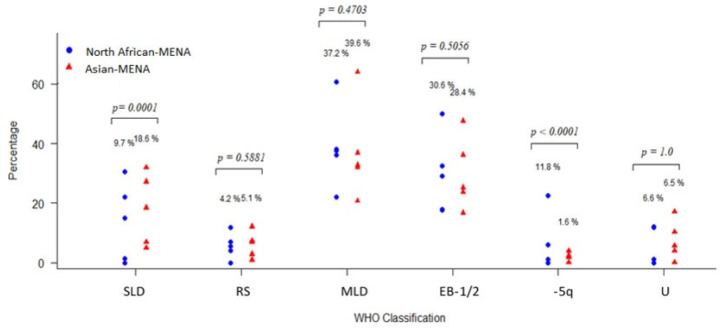
Comparison of MDS subtypes in the Asian MENA and North African MENA populations. MDS = myelodysplastic syndrome, MLD = multilineage dysplasia, SLD = single-lineage dysplasia, RS = ring sideroblasts, EB = excess blasts, Del5q = isolated del(5q), MDS-U = unclassifiable.

The WHO classification was available for 978 patients: 429 patients from Asian MENA and 549 from North African MENA populations. MDS with multilineage dysplasia (MDS-MLD) was the most commonly reported subclass in 38.2% of the total population, followed by refractory anemia with excess blasts-2 (RA-EB2) in 16.7%, single-lineage dysplasia (SLD) in 13.6%, RA-EB-1 in 13.1%, isolated deletion of 5q (del(5q)) in 7.4%, MDS unclassified (MDS-U) in 6.5%, and RA with ring sideroblasts (RARS) in 4.6% of patients. The proportion of WHO subclasses in the Asian and North African MENA regions are compared in Figure 4. The highest subclass in both regions was MDS-MLD, and chi squared tests for the equality of proportions indicated that the proportion of SLD and isolated del(5q) patients was significantly different between the two MENA populations (*p* < 0.0001; Figure 4).

**Figure 5:**
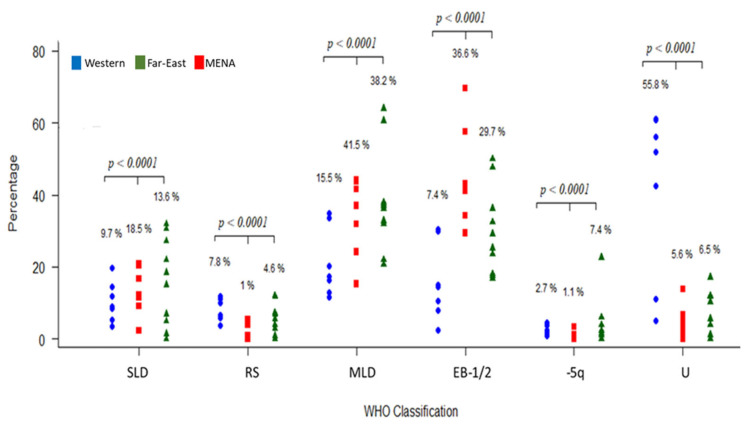
Comparison of the distribution of MDS subtypes between Western, Far East, and MENA populations. Western and Far East data were taken from [30,31,32,33,34,35,36,37,38,39,40,41]. MDS = myelodysplastic syndrome, MLD = multilineage dysplasia, SLD = single-lineage dysplasia, RS = ring sideroblasts, EB = excess blasts, −l5q = isolated del(5q), U = unclassifiable.

We next compared the proportion of MDS subtypes between MENA, Western, and Far East populations. There were significant differences in the proportion of patients belonging to the three groups for all MDS subtypes (*p* < 0.001). To examine the pairwise differences in subtype proportions between Western, Far East, and MENA populations, we applied pairwise proportion tests (pairwise.prop.test function) in R. There were significant differences in proportions between all group pairs except between Far East and MENA for the U (unclassifiable) subtype (Figure 5).

#### 3.3.5. Prognosis

**Figure 6:**
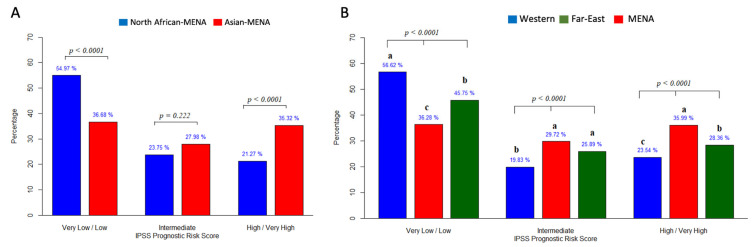
(**A**) Subgroup analysis of the IPSS categories in North African and Asian MENA populations. (**B**) Comparison of IPSS categories between Western, Far East, and MENA populations. We compared our MENA results with results from Western and Far East populations [15]. Letters show significant differences.

Figure 6A shows the IPSS-R prognostic risk scores reported in 730 patients from nine studies using three categories: very low/low, intermediate, and high/very high. In the MENA population, the very low/low category was the most common (45.8%). However, in subgroup analysis, there were significant differences in the very low/low category between the Asian MENA (36.68%) and North African MENA (54.97%) populations (*p* < 0.0001). For the higher high/very high category, the chi-squared test also indicated significant differences (*p* < 0.0001) between the proportions in the Asian MENA and North African MENA populations. 

We then compared the proportions of patients in different IPSS categories between MENA patients and Western and Far East populations (Western and Far East data from [15]; Figure 6B). A very low/low risk prognosis was most common in the Western and Far East populations (56.6% and 45.75%, respectively). However, the very low/low and high/very high populations were distributed equally in the MENA population: 36.28% and 35.99%, respectively. The chi-squared test results indicated a significant difference in proportions between Western, Far East, and MENA populations for the different risk score categories. The pairwise comparisons indicated that the proportion of patients was significantly different for all pairs for the various risk score categories except for between the Far East and MENA for the intermediate risk category.

#### 3.3.6. Karyotype

**Figure 7:**
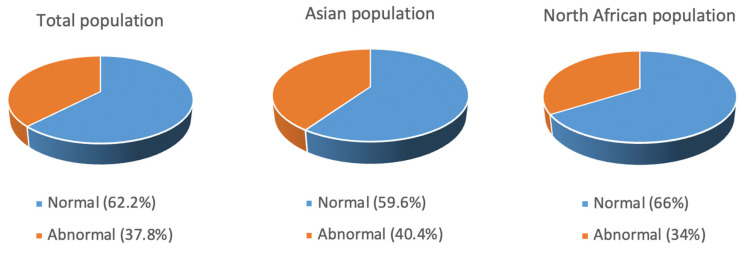
Karyotype distribution in the total population, Asian population, and North African population.

Eight studies reported karyotype data as normal or abnormal for 903 patients. In total, 562 patients (62.2%) had normal karyotypes, whereas 341 (37.8%) had abnormal karyotypes. When comparing the Asian and North African MENA populations, the Asian population had a higher prevalence of abnormal karyotypes than the African population, with these totaling 40.4% and 34.0%, respectively (Figure 7).

## 4. Discussion

To our knowledge, there have been no previous systemic reviews on MDS patients from the MENA region. To address this gap, in this systematic review, we summarized the results of thirteen articles from the MENA region describing the distinct clinical features of 1306 MDS patients. Seven studies were performed in the Asian MENA (732 patients), and six were conducted in the North African MENA (574 patients). We could not find a national registry for MDS in any MENA country. We analyzed the entire patient group as a whole and the Asia and the North African MENA countries separately. We found disparities in the characteristics of MDS patients from different MENA regions and with MDS patients from Western countries.

We found that MENA patients are younger at diagnosis and have a different MDS subtype distribution and prognosis. Most articles included in this review reported the age of onset of MDS in their patients as a mean value. Although some of these studies reported a high mean age, such as 65.7 years in a Morocco population [13] and 66 in an Iranian population [23], the mean age of all patients was 58.4 years. It has been reported that Asian countries such as India, China, Korea, and Japan have a younger mean age of onset of MDS than Western patients. For example, a study from China retrospectively examined the data on 502 patients collected between 2006 and 2011 and found that the mean age was 53 years, with almost 62% of patients younger than 60 years old [42]. By comparison, the mean age of MDS onset in Western countries is usually reported to be >10 years higher [43]. This difference between Asian and Western countries is well documented; however, in the MENA region, an age disparity was controversial. We found that the mean age was low in both the Asian and the North African MENA regions. The cause of these differences is not clear. However, genetic and environmental factors have been shown to play important roles in the pathoetiology of MDS. With respect to the gender distribution, our analysis revealed a similar male-to-female ratio as internationally reported, with an apparent male dominance. Only one study that we analyzed, which was from Morocco, favored female patients [25], but there were only 20 patients in this study and, in such a small cohort, bias is likely. 

The WHO definition of anemia is an Hb level < 12 g/dL for women and <13 g/dL for men [44]. The Hb level in all studies included in this systematic review was moderate to severe anemia (the maximum reported Hb was 10.3 g/dL in a study from Morocco [13]). The pooled mean Hb for all studies was 8.4, and the difference between Asian and North African MENA populations was not significant. This mean is higher than that reported for other Asian countries such as China (6.3 g/dL) [45] and India (6.84 g/dL) [46]. Similarly, mean platelet counts were comparable to the reported Western mean (158 × 10^3^/μL) [43]. However, the mean level in some Southeast Asian countries is much lower. For example, Ehsan et al. reported a mean value of 59.6 × 10^3^/μL in 46 patients from Pakistan [47]. Interestingly, in another study from Pakistan, the mean value was reported as 97 × 10^3^/μL in 178 patients [48]. Different diagnostic classifications and environmental and genetic factors may be responsible for this wide difference in Hb and platelet levels in MDS patients from Asia. 

Our analysis showed that MDS-MLD is the most common MDS subtype in the entire MENA region (38.2%) and in the Asian and North African areas (39.6% and 37.2%, respectively). This is similar to most published data from Western and Asian countries. For example, MDS-MLD is reported in 32.2% of patients in the USA, 44% in China, 36.9% in Pakistan, and 27.6% in Germany [49,50,51,52]. Moreover, the EB1 and EB2 subclasses were comparable in Western and Asian populations. However, the SLD subtype was significantly different between the MENA areas: 18.6% SLD in Asian MENA compared with 9.7% in the North African MENA region. Interestingly, a systemic review of 14 articles from Asian countries (China, Korea, and Japan) and Western countries (USA, Switzerland, Netherlands, Poland, Sweden, and Australia) reported an SLD distribution of 18.57% in Western countries and 9.7% in Asian countries, which is very similar to our findings in different MENA areas [15]. Del(5q) was present in a high percentage of North African MENA patients compared with Asian MENA patients. Generally, del(5q) is present in 1 to 5% of Western and Asian populations (14), which compares to our result of 11.8% of North Africa MENA MDS in our analysis. Other reports show a similarly high del(5q) percentage in this region. For example, Gmidene et al. reported that 13% of 224 MDS patients had the del(5q) subtype following the FAB classification [53]. It is well known that changing the classification criteria will change the subtype distribution [15]. The distribution of del(5q) needs further investigation in patients from the MENA region.

Cytogenetic abnormalities were slightly more common in the Asian MENA region than the North African MENA countries. In the entire group, abnormal cytogenetics were reported in 37.8% of MDS patients, similar to that reported in Greece (38.4%), Italy (39%), China (37.1), and India (34.6) [54,55,56] but lower than the 46.6% reported in patients from Pakistan and 47.5% in patients from India [48]. The IPSS-R prognostic risk score classifies patients into five groups. However, two of the articles included in this review presented the data in three groups, “very low/low”, “intermediate”, and “high/very high”, so we used this stratification to aid comparisons. The “very low/low” prognostic risk group was more common in North African compared with Asian MENA patients. Conversely, the “high/very high” group was more common in Asian MENA. A “high/very high” risk phenotype predominance has been reported in other Asian countries such as Japan and Pakistan [35,48]. Although the IPSS-R has been used as a prognostic scoring system for 15 years, it has its limitations. Patients characterized into IPSS risk categories can display different outcomes; for example, 25–30% of patients classified as having “low-risk” MDS die within two years of diagnosis [57]. Unfortunately, there were not enough data in the included articles to analyze survival rates. 

Our analyses have some limitations. MDS is often susceptible to referral bias, which results in less symptomatic cases being biopsied less and consequently being overlooked in epidemiological studies. There is also a lack of complete reported data about the age of onset, incidence rate, and gender distribution within different age groups in the MENA region. In some included studies, we inferred the number of participants, as the complete data were not provided. We excluded case reports from our analyses, which may have added bias to the study. Our study is also based on a relatively small sample size, which could affect the reliability of our findings, especially with respect to the IPSS-R, since it has shown to be prognostic at the large scale but not necessarily for local, smaller cohorts. Another limitation of the study is the lack of complete survival data.

## 5. Conclusions

Our findings establish that MENA patients are phenotypically and clinically different. This may reflect different underlying exposures, etiologies, and pathogenetics, impacting the management of this distinct population. Our study also suggests that severe MDS and an unfavorable prognosis are more common in the Asian MENA population than the North African MENA population. The clinical findings and classification scores were similar for the Asian MENA population and Southeast Asian population, suggesting shared genetic, immunologic, ethnic, and environmental factors that need further exploration. MDS is often associated with exposure to environmental risk factors including organic solvents, radiation, pesticides, and other pollutants, and the high prevalence and severity of MDS in Asian populations may be due to exposure to such stimuli. We also identified a lack of national registries of MDS in MENA countries and urge the region to take a more proactive approach to this problem. Future studies should address the relevance for clinical practice and if the international established prognostication and treatment schemes may apply to MENA countries.

## Figures and Tables

**Table 1:** Inclusion and exclusion criteria according to the PICOS statement.

	Included	Excluded
Classification	WPSS and IPSS	FAB
Population	MENA population	Other populations
Intervention	None	None
Comparator	Patient at presentation assessment	None
Study Design	Prospective and retrospective cohort report, case-control	RCTs, case reports
Primary Outcome	Disease severity at presentation	Irrelevant
Others	Published research articles	Unpublished data, review articles

Abbreviations: PICO: Population, Intervention, Comparator, Outcomes, and Study design. WPSS: World Health Organization (WHO) classification-based Prognostic Scoring System. IPSS: International Prognostic Scoring System. FAB: French-American-British. MENA: the Middle East and North Africa. RCTs: randomized controlled trials.

**Table 2:** Descriptive characteristics of the included studies (MDS = myelodysplastic syndrome, M = male, F = female, SD = standard deviation, NA = not available).

	Authors and Year of Study	Country	Number of Patients	Gender			Age Mean	SD
				M	F	Ratio (M:F)		
1	Talbi et al. (2019) [19]	Algeria	243	126	117	1.1	NA	NA
2	Bekadja et al. (2015) [20]	Algeria	85	55	30	1.8	53.3	19.9
3	El-Menshawy et al. (2021) [21]	Egypt	100	54	46	1.2	56	11.0
4	Elnahass et al. (2018) [22]	Egypt	50	29	21	1.4	56	12.03
5	Paridar et al. (2021) [23]	Iran	103	75	28	2.68	66	5.5
6	Otrock et al. (2015) [24]	Lebanon	58	29	29	1	71	10.0
7	Errahhali et al. (2016) [25]	Morocco	20	8	12	0.67	52.8	NA
8	El Maaroufi et al. (2020) [13]	Morocco	76	43	33	1.3	65.7	14.0
9	Udayakumar et al. (2017) [26]	Oman	36	18	18	1	63	12.7
10	AlMozain (2020) [12]	Saudi Arabia	82	50	32	1.6	50	18.0
11	Uyanik et al. (2015) [27]	Turkey	119	59	60	1	66.3	12.3
12	Demirkan et al. (2007) [28]	Turkey	113	76	37	2.1	64.7	12.4
13	Deviren et al. (2012) [29]	Turkey	221	132	89	1.5	48.1	16.7
Total for North African MENA			574	315	259	1.3	57.3	
Total for Asian MENA			732	439	293	1.5	59.0	
Overall total			1306	754	552	1.4	58.4	

**Table 3:** Cytopenia and bone marrow blast values at diagnosis.

Clinical Parameter		All Patients (Total *n* = 359) *n* (%)	North African MENA (Total *n* = 264) *n* (%)	Asian MENA (Total *n* = 95) *n* (%)
Cytopenia	None	1 (0.3)	0	1 (1.1)
	Unicytopenia	106 (38.2)	85 (32.2)	52 (54.7)
	Bicytopenia	99 (27.6)	84 (31.8)	15 (15.8)
	Pancytopenia	122 (34)	95 (36)	27 (28.4)
		**All Patients (Total *n* = 191) *n* (%)**	**North African MENA (Total *n* = 124) *n* (%)**	**Asian MENA (Total *n* = 67) *n* (%)**
Bone marrow blasts	<5%	134 (70.2)	90 (72.5)	44 (65.6)
	5–9%	35 (18.3)	21 (17)	14 (21)
	10–19%	22 (11.5)	13 (10.5)	9 (13.4)

## Data Availability

No new data were created or analyzed in this study. Data sharing is not applicable to this article.

## Supplementary Materials

The following supporting information can be downloaded at: https://www.mdpi.com/article/10.3390/jcm12082832/s1, Table S1. PRISMA 2020 Checklist.
